# Financial judgment determination in adults with ADHD

**DOI:** 10.1007/s00702-021-02323-1

**Published:** 2021-03-12

**Authors:** Janneke Koerts, Dorien F. Bangma, Anselm B. M. Fuermaier, Christian Mette, Lara Tucha, Oliver Tucha

**Affiliations:** 1 Department of Clinical and Developmental Neuropsychology, University of Groningen, Grote Kruisstraat 2/1, 9712 TS Groningen, The Netherlands; 2 Department of Psychology, University of Amsterdam, Amsterdam, The Netherlands; 3 Department of Social Work and Education, Protestant University of Applied Sciences Bochum, Bochum, Germany; 4 LVR Hospital Essen, Essen, Germany; 5 Department of Psychiatry and Psychotherapy, Faculty of Medicine, University of Duisburg-Essen, Duisburg-Essen, Germany; 6 Department of Psychiatry and Psychotherapy, University Medical Center Rostock, Rostock, Germany; 7 Department of Psychology, Maynooth University, National University of Ireland, Maynooth, Ireland

**Keywords:** Financial capability, Financial decision-making, Financial judgment, ADHD

## Abstract

ADHD has a debilitating influence on everyday functioning, including the capability to make financial decisions. The capability to make financial decisions is a multidimensional construct that includes financial knowledge, financial judgment, financial performance and related contextual factors. So far, the majority of studies in adults with ADHD focused on financial performance, while the other aspects of financial capability were less explored. The current study aims to partly bridge this gap by examining the ability of financial judgment in adults with ADHD. Thirty-nine adults with ADHD and 83 adults without ADHD were included. All participants were assessed with the Financial Competence Assessment Inventory (FCAI) and Financial Decision-Making Interview (FDMI) which both assess the four abilities of financial judgment, i.e., understanding, appreciation, reasoning and communication. The results show that adults with ADHD, compared to adults without ADHD, obtained significantly lower scores on understanding (according to the FCAI and FDMI). Furthermore, adults with ADHD showed a significantly lower appreciation, reasoning and communication (according to the FCAI) than adults without ADHD. In conclusion, adults with ADHD have difficulties with financial judgment especially with the ability to understand information that is relevant for a financial situation or transaction. Furthermore, adults with ADHD were found to have problems with appreciating, reasoning and communicating about practical information that partly relates to their own financial situation (as assessed with the FCAI). A careful assessment of financial capability in adults with ADHD, therefore, appears warranted in clinical practice.

## Introduction

Attention-deficit/hyperactivity disorder (ADHD) is a common condition with a prevalence of approximately 3.4% in adulthood (Fayyad et al. 2007). In the last 2 decades, numerous studies showed that ADHD has a debilitating influence on many aspects of everyday functioning, including social, occupational and educational functioning (Barkley et al. 2008; Daley and Birchwood 2010; Michielsen et al. 2015; Nijmeijer et al. 2008). Recently, the capability to make financial decisions was added to this list (Altszuler et al. 2016; Bangma et al. 2019, 2020; Barkley and Fischer 2010; Das et al. 2012; Pelham et al. 2020). The capability to make financial decisions is crucial for independent and autonomous living and has been defined as the ability to manage one’s finances in a way that meets one’s best interests (Appelbaum et al. 2016). This capability encompasses relatively simple decisions, such as buying a loaf of bread at the bakery, as well as rather complex decisions, including taking out health insurance or buying a house. According to the conceptual model of financial capability of Appelbaum and colleagues (2016), financial capability includes financial competence and financial performance. Financial competence refers to financial skills that can be assessed in a controlled setting and can be subdivided into financial knowledge and financial judgment. Financial knowledge concerns declarative as well as procedural knowledge that is needed for the management of finances, including knowledge about the value of currency and online banking procedures. Financial judgment includes four abilities that are needed to make financial decisions that meet one’s best interest, i.e., understanding, appreciation, reasoning and communication. These four abilities are also considered to be the legal criteria for competency (Grisso and Appelbaum 1995). An adequate financial performance finally refers to “sufficient financial competence (knowledge and judgment) to implement financial decisions in the real world, that is, the presence of sufficient cognitive, perceptual, affective, communicative, and interpersonal abilities to manage […] one’s benefits” (Appelbaum et al. 2016, p. 6). Financial competence is, therefore, not always similar to financial performance in everyday life since many contextual factors (e.g., symptoms of depression, social support or the tendency to buy on impulse) can improve or lessen an individual’s financial performance.

So far, only a few studies examined the capability to make financial decisions in adults with ADHD. These studies indicate that adults with ADHD are more often financially dependent on others and report more often problems with impulse buying, exceeding credit card limits, a lower saving-income ratio, and problems with saving money than healthy individuals (Altszuler et al. 2016; Barkley et al. 2006, 2008). Furthermore, adults with ADHD were found to have lower income levels than healthy individuals (Biederman and Faraone 2006), and symptoms of hyperactivity-impulsivity and inattention were associated with self-reported financial problems (Altszuler et al. 2016; Barkley and Fischer 2010; Das et al. 2012). These results are corroborated by two studies of our group that used several standardized, performance-based tests and self-report questionnaires that focused on financial knowledge, financial judgment, financial performance and related contextual factors (Bangma et al. 2019, 2020). It was found that adults with ADHD showed significantly lower performances on tests of financial knowledge and judgment than adults without ADHD. Furthermore, compared to adults without ADHD, adults with ADHD showed difficulties in making decisions referring to the future and reported more often to experience impulse buying and the use of a spontaneous or avoidant decision-making style.

The capability to make financial decisions thus appears to be decreased in adults with ADHD. However, so far, the majority of studies in adults with ADHD focused on financial performance, while the other aspects of financial capability (i.e., financial knowledge and financial judgment) were less explored. The current study aims to partly bridge this gap by specifically examining the ability of financial judgment in adults with ADHD. The reasons for focusing on financial judgment are that this concept (1) plays an essential role in the evaluation of an individual’s financial capability (Appelbaum et al. 2016), (2) encompasses abilities (i.e., understanding, appreciation, reasoning, and communication) that are considered to be the legal criteria for (financial) competence (Grisso and Appelbaum 1995) and (3) is assessed by many tests that focus on (aspects of) financial competence (Engel et al. 2016). Learning more about this ability in adults with ADHD will assist the development of tailored financial support which appears needed in this group. In the present study, financial judgment will be assessed with the Financial Decision-Making Interview (FDMI, Bangma et al. 2017) and the Financial Competence Assessment Inventory (FCAI, Kershaw and Webber 2008). The FDMI assesses financial judgment using two different vignettes, each including a hypothetical abstract financial problem, i.e., (1) repairing or selling a car and (2) selling and buying a house, about which open-ended questions are asked, e.g., ‘What does Harry have to make a decision about and why?’ or 'What are Harry's options? What are the (dis)advantages of this decision?' (Bangma et al. 2017). The FCAI, on the other hand, assesses financial judgment using practical, concrete, everyday assignments that partly focus on the financial situation of the participant. For example, 'Tell me about your income. Where does your money (to pay bills, etc.) come from, and how much do you receive each pay period?' or 'Do you normally keep money on one side to pay unexpected bills, such as, your car breaking down/needing a taxi in an emergency?' (Kershaw and Webber 2008). In addition to studying the performance of adults with ADHD on financial judgment tests, the construct validity of both tests will be determined. More specifically, by conducting within-person comparisons of and correlations between the performances on (different parts of) these tests, the convergent validity (i.e., the degree to which measures of the same concept are related) of financial judgment will be determined. In addition, the divergent validity (i.e., the degree to which measures of different concepts are related) will be evaluated by examining the associations between the FCAI, FDMI and tests that assess financial constructs other than financial judgment.

## Methods

### Participants

Forty-eight adults with ADHD and 85 healthy controls were included in the present study. Adults with ADHD were recruited at the Department of Psychiatry and Psychotherapy of the University of Duisburg-Essen, Germany, and were diagnosed according to the criteria of ADHD as described in the Diagnostic and Statistical Manual of Mental Disorders, 5^th^ edition (American Psychiatric Association 2013). The diagnostic procedure included semi-structured interviews for ADHD psychopathology (i.e., the Wender–Reimherr Interview (Retz-Junginger et al. 2017) and the Essen-Interview-for-school-days-related-biography (Grabemann et al. 2017)), self-report questionnaires for current and retrospective symptoms (i.e., the Wender Utah Rating Scale—Childhood (WURS-K) and the ADHD self-report scale (ADHD-SR, Rösler et al. 2008)) and objective measures (e.g., school reports, reports of academic and/or occupational failures) which were obtained from multiple informants (e.g., partner and employer). Adults with ADHD were free of stimulants during the 48 h prior to assessment. Healthy controls were recruited via word-of-mouth, contacts of the researchers and social media and also completed the WURS-K and ADHD-SR. Healthy controls were excluded when scoring above the cut-off on both of these questionnaires (i.e., WURS-K ≧ 30 and ADHD-SR ≧ 18, Rösler et al. 2008), whereas adults with ADHD were excluded when scoring below the cut-off on both questionnaires (i.e., WURS-K < 30 and ADHD-SR < 18, Rösler et al. 2008). Other exclusion criteria that applied to all participants were the presence of neurological or psychiatric conditions other than ADHD that were assessed by means of self-report prior to assessment in healthy controls or during the clinical assessment for ADHD in adults with ADHD. Adults with ADHD who had comorbid disorders (i.e., substance dependency, depressive disorder, adjustment disorder or personality disorder) that are typical for this condition were, however, not excluded. Finally, since within-person comparisons and correlations are an important part of the analyses in the present manuscript, participants were excluded when data was missing for one or more of the tests described below. The latter resulted in the exclusion of data of nine adults with ADHD and two healthy controls.

The demographic and clinical characteristics of both groups (ADHD *n* = 39; healthy controls *n* = 83) are described in Table 1. No differences were found between groups regarding age (*t* = − 1.60; *p* = 0.11), sex (*X*^2^ = 1.66; *p* = 0.20) and the number of years of education (*t* = − 0.47; *p* = 0.64). As expected, adults with ADHD reported significantly more childhood and current symptoms of ADHD on the WURS-K (*t* = − 12.23; *p* < 0.001) and ADHD-SR (*t* = − 16.37; *p* < 0.001), respectively, compared to healthy controls.

**Table 1:** Demographic and clinical characteristics of adults with (*n* = 39) and without ADHD (*n* = 83)

	Adults with ADHD	Adults without ADHD
	M (SD)	M (SD)
Age in years	36.5 (10.4)	32.7 (13.0)
Education in years	16.8 (3.2)	16.5 (3.5)
Sex: % female	43.5	56.1
Work-status		
% Full-time	53.8	36.1
% Part-time	23.1	10.8
% Unemployed	10.3	7.2
% Student	2.6	38.6
% Other	10.3	7.2
WURS-K	43.3 (13.6)	13.2 (8.6)
ADHD-SR	35.7 (8.1)	11.3 (7.3)
Presentation of ADHD		
% Combined	61.5	–
% Inattentive	23.1	–
% Hyperactive/impulsive	0	–
% Not specified	15.4	–
Comorbidities		
% Adjustment disorder	12.8	–
% Depressive disorder	15.4	–
% Personality disorder	10.3	–
% Substance dependency	7.7	–

*WURS-K* Wender Utah Rating Scale—Childhood, *ADHD-SR* ADHD self-report scale

### Materials

#### Financial judgment

The Financial Competence Assessment Inventory (FCAI, Kershaw and Webber 2008) is a test of financial competence that assesses six different components: (1) everyday financial abilities, (2) financial judgment, (3) estate management, (4) cognitive functioning related to financial tasks, (5) debt management, and (6) support resources. The FCAI consists of 38 items which include practical, concrete, everyday assignments which partly focus on the financial situation of the participant. Items of the FCAI are scored on a 5-point scale ranging from 0 (i.e., little or no awareness) to 4 (i.e., complete understanding), with the exception of six items which are scored as yes/no (i.e., 0/1). Even though the FCAI includes the component ‘financial judgment’, it is important to mention that this component is dissimilar to what is meant with financial judgment in the conceptual model of Appelbaum and colleagues, i.e., four abilities that are needed to make financial decisions that meet one’s best interest (understanding, appreciation, reasoning and communication, Appelbaum et al. 2016). Instead, the FCAI allows the conversion of seventeen items to scores on these four abilities: understanding (maximum score is 20), appreciation (maximum score is 20), reasoning (maximum score is 20) and expressing a choice (maximum score is 8).

The Financial Decision-Making Interview (FDMI, Bangma et al. 2017) assesses the four abilities of financial judgment as defined in the conceptual model of Appelbaum and colleagues (2016). For this purpose, two vignettes are used, each of which includes a complex, relatively abstract hypothetical financial problem (i.e., (1) repairing or selling a car and (2) selling and buying a house). Each vignette is presented orally and in writing to the participant. During a semi-structured interview, participants are asked several (open-ended) questions related to the four abilities of financial judgment (e.g., ‘What could the protagonist choose to do?’, ‘What do you think the protagonist should do?’, ‘Whom does this choice affect?’, ‘Will it affect anyone else?’). Each answer receives a score of 0, 1 or 2 depending on the level of completeness. For each vignette, scores are calculated for understanding (maximum score is 2), appreciation (maximum score is 2), reasoning (maximum score is 2) and communication (maximum score is 2). Furthermore, the scores for each vignette can be summed up to determine total scores for each ability (maximum score per ability is 4).

#### Financial constructs other than financial judgment

The Temporal Discounting Task (TDT, Bangma et al. 2017) examines the ability to make decisions with implications for the future. Temporal discounting refers to the phenomenon that the subjective value of money decreases over time (Green et al. 1994), i.e., €100 today has a higher subjective value than the same amount after one year. The TDT consists of eighteen hypothetical scenarios. During each scenario, participants have to indicate the lowest amount of money they would accept after a short time period compared to a high amount of money after a longer time period. For example, "Which amount of money would you accept after 1 week, instead of €1000 after 1 year". Three amounts of money that participants can receive after a longer time period are used (i.e., €100, €500 and €1000) which are combined with six different time intervals: (1) today vs. 1 week, (2) today vs. 1 month, (3) today vs. 1 year, (4) 1 week vs. 1 month, (5) 1 week vs. 1 year and (6) 1 month vs. 1 year. For each scenario, the answer is converted to the percentage of the amount of money the participant can receive after a longer time period. Subsequently, an average score is calculated for all 18 scenarios combined with lower percentages indicating a stronger temporal discounting tendency.

The Iowa Gambling Task (IGT, Bechara et al. 1994) is a computerized test that is used to assess emotional decision-making (Buelow and Suhr 2009). During the IGT, participants have to choose cards from four decks that are each associated with certain gains and losses. Two disadvantageous decks lead to relatively high gains but also to high losses, while two advantageous decks are associated with relatively small gains but also small losses. In total, participants have to complete 100 trials. A netscore over 100 trials is calculated by subtracting the number of times a disadvantageous deck was chosen from the number of times an advantageous deck was chosen.

The Competence in Decision Rules (CDR, Bangma et al. 2017) assesses the ability to make decisions based on specific rules. Ten scenarios of increasing complexity are presented. During each scenario participants have to decide which television(s) out of five televisions they would buy when applying different decision rules. The five televisions differ in technical specifications (e.g., sound quality) but are equally priced. To select the correct television(s), participants need to evaluate the specifications of all televisions and need to determine which one(s) is/are the best choice given a specific rule. The total number of correctly completed scenarios is rated (maximum score is 10).

### Ethics statement and procedure

This study was approved by the Ethical Committee of the Faculty of Medicine of the University of Duisburg-Essen, Germany. All participants signed an informed consent prior to study inclusion. All participants were assessed individually and received as many breaks as needed.

### Statistical analyses

#### Between-group comparisons

Performances of adults with and without ADHD on all financial tests were compared. No serious violation of normality was observed for the scores on the FCAI, CDR and IGT and performances were compared using *t*-tests. The scores on the TDT were normally distributed after an arcsine transformation for percentage data (i.e., 2*arcsine√*X*_i_/100; Cohen et al. 2003) and were also compared using a *t*-test. Finally, since the performances on the different subscales of the FDMI were measured on ordinal level, Mann–Whitney *U* tests were used for the comparison of performances on these variables. Effect sizes (including 99% confidence intervals) were calculated (by means of the effect size calculator of www.psychometrica.de) for all comparisons and were interpreted as small (*d* = 0.20), medium (*d* = 0.50) and large (*d* = 0.80). Differences were considered significant when *p* ≦ 0.01 to control for inflation of type-I errors.

#### Convergent validity

To determine the convergent validity of the vignettes of the FDMI, Wilcoxon signed-ranked tests were used to compare the performances on both vignettes. These within-group comparisons were conducted in both groups combined (i.e., adults with and without ADHD). Differences were considered significant when *p* ≦ 0.01 and effect sizes were determined (by means of the effect size calculator of www.psychometrica.de). In case significant differences are found, the analyses will be repeated for both groups separately. Furthermore, Kendall's tau correlations were calculated in both groups combined between the performances on the FCAI and FDMI (i.e., both vignettes combined). According to the criteria of Cohen (1988), correlations of 0.10 are classified as low, correlations of 0.30 as medium and correlations of 0.50 as high. A correlation of 0.50 or higher, which indicates a shared variance of at least 25%, was used as an indication for sufficient convergent validity (Huizinga et al. 2020). Finally, the performances on the FCAI were compared to normative data as published in the manual (Kershaw and Webber 2008) and the number of participants scoring ≦ 1 SD below the mean was rated for each subscale. Also, the number of participants who obtained a score of ≦ 1 on the four subscales of the FDMI for both vignettes separately was determined. Subsequently, Cohen's kappa was calculated in both groups combined to determine the agreement for understanding, appreciation, reasoning and communication between the FCAI and FDMI, and between both vignettes of the FDMI. Agreement was obtained when the following scores were observed:

- A score of > 1SD below M on the FCAI and score > 1 on vignette 1 of the FDMI *or* a score of ≦ 1 SD below M on the FCAI and a score ≦ 1 on vignette 1 of the FDMI;
- A score of > 1SD below M on the FCAI and score > 1 on vignette 2 of the FDMI *or* a score of ≦ 1 SD below M on the FCAI and a score ≦ 1 on vignette 2 of the FDMI;
- A score > 1 on vignette 1 of the FDMI and score > 1 on vignette 2 of the FDMI *or* a score ≦ 1 on vignette 1 of the FDMI and score ≦ 1 on vignette 2 of the FDMI.

Cohen's kappa was interpreted as no agreement (i.e., κ ≦ 0), no to slight agreement (i.e., κ = 0.01–0.20), fair agreement (i.e., κ = 0.21–0.40), moderate agreement (i.e., κ = 0.41–0.60), substantial agreement (i.e., κ = 0.61–0.80) and almost perfect agreement (i.e., κ = 0.81–1.00; McHugh 2012).

#### Divergent validity

To explore the divergent validity of the tests of financial judgment, Kendall's tau correlations were calculated in both groups combined between the performances on the FCAI, FDMI (i.e., both vignettes combined), CDR, IGT and TDT. A correlation of 0.30 or lower, with a maximum shared variance of 9%, was used as an indication of sufficient divergent validity (Huizinga et al. 2020).

## Results

### Between-group comparisons

Adults with ADHD showed significantly lower performances on the understanding, appreciation, reasoning and communication subscales of the FCAI compared to adults without ADHD (medium to large effect sizes; Table 2). On the first as well as on the second vignette of the FDMI, adults with ADHD also obtained significantly lower scores on understanding than adults without ADHD (medium effect sizes), while no significant differences were found between groups regarding appreciation, reasoning and communication of both vignettes. Finally, significant group differences were found for the TDT on which adults with ADHD scored significantly lower than adults without ADHD (large effect size). No significant differences were found between groups for the IGT and CDR (Table 2).

**Table 2:** Performances of adults with (*n* = 39) and without ADHD (*n* = 83) on tests of financial judgment and financial performance

	Adults with ADHD	Adults without ADHD	Statistic	*p*	*d* [99%CI]
	M (SD)	M (SD)			
Financial judgment					
FCAI understanding	14.5 (3.0)	16.7 (2.5)	*t* = 4.2	< 0.001*	0.82 [0.30 to 1.34]
FCAI appreciation	15.7 (2.7)	17.4 (2.6)	*t* = 3.3	0.001*	0.65 [0.13 to 1.16]
FCAI reasoning	14.4 (2.4)	17.0 (2.7)	*t* = 5.3	< 0.001*	1.00 [0.47 to 1.53]
FCAI communication	4.9 (1.7)	6.7 (1.3)	*t* = 6.0	< 0.001*	1.25 [0.71 to 1.79]
FDMI vignette 1					
FDMI understanding	1.5 (0.5)	1.9 (0.3)	*z* = − 4.7	< 0.001*	0.67 [0.15 to 1.18]
FDMI appreciation	1.6 (0.6)	1.6 (0.5)	*z* = − 0.3	0.746	0.05 [− 0.45 to 0.55]
FDMI reasoning	1.7 (0.6)	1.9 (0.3)	*z* = − 2.5	0.011	0.26 [− 0.24 to 0.76]
FDMI communication	1.9 (0.3)	1.9 (0.3)	*z* = − 0.6	0.571	0.05 [− 0.45 to 0.55]
FDMI vignette 2					
FDMI understanding	1.4 (0.6)	1.8 (0.5)	*z* = − 2.8	0.005*	0.43 [− 0.07 to 0.93]
FDMI appreciation	1.5 (0.6)	1.8 (0.5)	*z* = − 2.2	0.029	0.32 [− 0.18 to 0.82]
FDMI reasoning	1.8 (0.5)	1.9 (0.3)	*z* = − 0.4	0.714	0.04 [− 0.46 to 0.54]
FDMI communication	1.8 (0.5)	1.9 (0.3)	*z* = − 1.6	0.117	0.16 [− 0.34 to 0.66]
Financial performance					
TDT	75.0 (19.5)	87.3 (11.1)	*t* = 4.5	< 0.001*	0.86 [0.34 to 1.38]
IGT	18.6 (42.6)	14.4 (36.0)	*t* = − 0.5	0.600	− 0.11 [− 0.61 to 0.39]
CDR	7.0 (2.1)	7.6 (2.0)	*t* = 1.5	0.131	0.30 [− 0.20 to 0.80]

*FCAI* Financial Competence Assessment Inventory, *FDMI* Financial Decision-Making Interview, *TDT* Temporal Discounting Task, *IGT* Iowa Gambling Task, *CDR* Competence in Decision Rules, *CI* confidence interval

*p ≦ 0.01

### Convergent validity

In Table 3, the within-group comparison of the performances of both groups combined on vignette 1 and 2 of the FDMI are presented. No significant differences were found between the scores on understanding, appreciation, reasoning and communication of both vignettes. The correlations between the FCAI and FDMI (i.e., both vignettes combined and both groups combined) were, however, all below the cut-off for sufficient convergent validity (i.e., a correlation of 0.50 or higher; FCAI understanding and FDMI understanding: Kendall's tau = 0.18, *p* = 0.018, FCAI appreciation and FDMI appreciation: Kendall's tau = 0.15, *p* = 0.050, FCAI reasoning and FDMI reasoning: Kendall's tau = 0.22, *p* = 0.004, FCAI communication and FDMI communication: Kendall's tau = 0.14, *p* = 0.072).

**Table 3:** Within-group comparison of performances on the vignettes of the FDMI (*n* = 122)

	M (SD)	*z*	*p*	*d*
Vignette 1: understanding	1.8 (0.4)	− 1.9	0.052	0.36
Vignette 2: understanding	1.7 (0.5)			
Vignette 1: appreciation	1.6 (0.6)	1.0	0.320	0.18
Vignette 2: appreciation	1.7 (0.5)			
Vignette 1: reasoning	1.9 (0.4)	− 0.2	0.841	0.04
Vignette 2: reasoning	1.9 (0.4)			
Vignette 1: communication	1.9 (0.3)	− 1.3	0.197	0.24
Vignette 2: communication	1.9 (0.4)			

*CI* confidence interval

Finally, the scores on the FCAI and on both vignettes of the FDMI were classified as ≦ 1SD below M/ > 1 SD below M compared to normative data or as a score of ≦ 1/score of > 1, respectively. Subsequently, Cohen's kappa was calculated to determine the level of agreement for understanding, appreciation, reasoning and communication between the FCAI and FDMI and between both vignettes of the FDMI. The percentage of participants that was classified similarly according to the FCAI and FDMI (i.e., as > 1SD below M and score > 1 or as ≦ 1 SD below M and score ≦ 1, respectively) ranged between 49.2% and 60.7% for vignette 1 (Table 4) which indicated no to slight agreement for understanding (к = 0.10), appreciation (к = 0.03), reasoning (κ = 0.12) and no agreement for communication (κ ≦ 0.001). Similar results were found for the FCAI and vignette 2 of the FDMI for which the percentage of participants that was classified similarly ranged between 54.1% and 65.7% (Table 4) with no to slight agreement for understanding (κ = 0.19), appreciation (κ = 0.18), reasoning (κ = 0.05) and communication (κ = 0.09). Table 5 presents the comparison of the classifications according to both vignettes of the FDMI. The percentage of participants that was classified similarly (i.e., as having a score of > 1 for vignette 1 as well as for vignette 2 or as having a score of ≦ 1 for vignette 1 and 2) ranged between 66.4 and 88.5% and Cohen's kappa indicated fair agreement for understanding (κ = 0.26), appreciation (κ = 0.23), reasoning (κ = 0.21) and communication (κ = 0.36).

**Table 4:** Classification of scores on the FCAI versus FDMI vignette 1 and FDMI vignette 2 (*n* = 122)

	*FDMI Vignette 1*							
	Understanding		Appreciation		Reasoning		Communication	
	% > 1	% ≦ 1	% > 1	% ≦ 1	% > 1	% ≦ 1	% > 1	% ≦ 1
FCAI understanding								
% > 1 SD below M	49.2	13.1						
% ≦ 1 SD below M	26.2	11.5						
FCAI appreciation								
% > 1 SD below M			47.1	24.0				
% ≦ 1 SD below M			18.2	10.7				
FCAI reasoning								
% > 1 SD below M					50.8	4.1		
% ≦ 1 SD below M					36.9	8.2		
FCAI communication								
% > 1 SD below M							45.1	4.1
% ≦ 1 SD below M							46.7	4.1

	*FDMI Vignette 2*							
	Understanding		Appreciation		Reasoning		Communication	
	% > 1	% ≦ 1	% > 1	≦ 1	> 1	≦ 1	> 1	≦ 1
FCAI understanding								
% > 1 SD below M	46.7	15.6						
% ≦ 1 SD below M	21.3	16.4						
FCAI appreciation								
% > 1 SD below M			53.7	17.4				
% ≦ 1 SD below M			16.5	12.4				
FCAI reasoning								
% > 1 SD below M					48.4	6.6		
% ≦ 1 SD below M					37.7	7.4		
FCAI communication								
% > 1 SD below M							45.9	3.3
% ≦ 1 SD below M							42.6	8.2

*FCAI* Financial Competence Assessment Inventory, *FDMI* Financial Decision-Making Interview

**Table 5:** Classification of scores on vignette 1 versus vignette 2 of the FDMI (*n* = 122)

	*Vignette 2*												
	Understanding		Appreciation			Reasoning				Communication			
	% > 1	% ≦1	% > 1		% ≦1	% > 1			% ≦1	% > 1			% ≦1
Vignette 1													
Understanding													
% > 1	56.6	18.9											
% ≦ 1	11.5	13.1											
Appreciation													
% > 1				50.8	14.8								
% ≦ 1				18.9	15.6								
Reasoning													
% > 1							77.9	9.8					
% ≦ 1							8.2	4.1					
Communication													
% > 1											84.4	7.4	
% ≦ 1											4.1	4.1	

### Divergent validity

All correlations between the FCAI, TDT, IGT and CDR indicated sufficient divergent validity of the FCAI (i.e., all correlations were below 0.30, Table 6). Similar results were found for the FDMI (Table 6).

**Table 6:** Divergent validity: Correlations between FCAI, FDMI (both vignettes combined), TDT, IGT and CDR (*n* = 122)

	TDT	IGT	CDR
FCAI understanding	0.20	− 0.11	0.15
FCAI appreciation	0.21	− 0.01	0.03
FCAI reasoning	0.19	0.03	0.09
FCAI communication	0.25	0.02	0.17
FDMI understanding	0.10	0.00	0.20
FDMI appreciation	0.05	0.06	0.00
FDMI reasoning	− 0.02	− 0.11	− 0.04
FDMI communication	0.07	− 0.04	0.18

*FCAI*: Financial Competence Assessment Inventory, *FDMI* Financial Decision-Making Interview, *TDT* Temporal Discounting Task, *IGT* Iowa Gambling Task, *CDR* Competence in Decision Rules

## Discussion

The aim of the current study was to examine the ability of financial judgment in adults with ADHD. Financial judgment is an important aspect of the capability to make financial decisions (Appelbaum et al. 2016) and encompasses abilities (i.e., understanding, appreciation, reasoning, and communication) that are considered to be the legal criteria for (financial) competence (Grisso and Appelbaum 1995). Consistent with previous research (Altszuler et al. 2016; Barkley and Fischer 2010; Barkley et al. 2006, 2008; Biederman and Faraone 2006; Das et al. 2012), it was found that adults with ADHD, compared to adults without ADHD, obtained significantly lower scores on tests of financial judgment. Furthermore, adults with ADHD showed a stronger temporal discounting tendency than adults without ADHD. Previous studies demonstrated that adults with ADHD have a reduced information processing capacity (Roberts et al. 2012) and impairments in working memory (Alderson et al. 2013), speed of information processing (Wiig and Nielsen 2012), vigilance/sustained attention (Tucha et al. 2009, 2015), selective attention (Tucha et al. 2008) and arithmetic abilities (Frazier et al. 2004), all which may underly difficulties with understanding financial information. Also, the comorbidities (e.g., depressive disorders or substance dependency) that were present in some of the adults with ADHD might partly explain the differences that were found regarding financial judgment and temporal discounting between adults with and without ADHD. The most consistent finding of the present study was, however, that adults with ADHD have difficulties in understanding financial information, as indicated by significantly lower performances on the FCAI and the vignettes 1 and 2 of the FDMI compared to adults without ADHD. Understanding within the context of financial judgment refers to the competence to understand information that is relevant for a financial situation or transaction (Appelbaum et al. 2016; Marson 2016), an ability that is crucial for everyday functioning and autonomy. In this context, it is important to note that the FCAI and FDMI assess understanding at different levels. The FCAI assesses understanding at a rather practical/concrete level and scores are based on the sum of scores on questions such as "Do you receive any bills? If there are bills in the mail, how do you recognize them?", "This is a typical household bill. Please read the bill and tell me who it is from and how much is due? When is this bill due for paying?", "If there was not sufficient money in the account to cover the cheque, but there was an overdraft facility on the account, would the bank automatically pay it? Why is that?", "If this bill was paid by credit-card, would it cost more money? Why is that, how does a credit-card work?" and "Tell me what assets are?" (Kershaw and Webber 2008). The FDMI, on the other hand, makes use of hypothetical vignettes that are more abstract. For example, a couple with a baby on the way considers selling their current house. However, houses such as theirs sell rather slow and they, therefore, decided to put their house on the market for a relatively low price. Should they now already buy a new house? The bank calculated that they can afford to pay the mortgages of two houses for a maximum period of 11 months. What should they do? (Bangma et al. 2017). Understanding in the context of the FDMI is subsequently evaluated by asking "What options does this couple have?" and "Imagine they buy the new house now, while they did not sell their old house yet. What are the (dis)advantages of this decision?" The results of the present study thus indicate that adults with ADHD have difficulties with understanding practical, concrete, everyday information as well as with understanding more abstract information in a financial context compared to adults without ADHD. Difficulties which can result in serious financial problems such as misunderstanding financial information on, for example, bills, insurance websites or leaflets or official documentation of the bank.

Regarding the other three abilities of financial judgment, i.e., appreciation, reasoning and communication, somewhat inconsistent results were found. According to the FCAI, adults with ADHD have more difficulties with appreciation, reasoning and communication than adults without ADHD. However, no differences were found for these three abilities of financial judgment according to the FDMI. This inconsistency between results might be explained by the different levels at which the FCAI and FDMI assess financial judgment. As already mentioned above, the FCAI uses a more practical, concrete, everyday approach, while the FDMI uses vignettes which are more abstract. In addition, the FCAI partly requires participants to evaluate their own personal financial situation, while the FDMI asks participants to discuss a hypothetical situation of another person. For example, appreciation during the FCAI is, amongst others, evaluated by checking if a participant knows where their money goes each month, reasoning is, amongst others, rated by determining whether a participant can budget and calculate their own account balance, and communication is, amongst others, evaluated by asking whether a participant can formulate long-term financial goals (Kershaw and Webber 2008). These results taken together thus indicate that, compared to adults without ADHD, adults with ADHD have difficulties with appreciating, reasoning and communicating about practical, everyday information that partly relates to their own financial situation, while they appear to have no problems with appreciation of and reasoning and communication about abstract, hypothetical financial information. These results are consistent with the fact that previous studies reported that adults with ADHD often have practical everyday financial problems such as being more often financially dependent on others, reporting more often problems with impulse buying, exceeding credit card limits, a lower saving-income ratio, and problems with saving money (Altszuler et al. 2016; Barkley et al. 2006, 2008). Since these practical everyday financial difficulties can result in serious financial problems, tailored teaching and coaching programs are urgently needed that support adults with ADHD in dealing with their financial matters. It should be pointed out, however, that even though adults with ADHD appear to have problems with the legal abilities of (financial) competency (Grisso and Appelbaum 1995), i.e., with understanding, appreciation, reasoning and communication, especially in practical, everyday situations, that these findings do not justify legal decisions regarding the financial capability of adults with ADHD. As described by Kane and colleagues (2021), there is a translational gap between how these four abilities should be understood and how they should be applied in a court of law. Additionally, as also described in the conceptual framework of Appelbaum and colleagues (2016), financial capability does not only include financial judgment but also financial knowledge, financial performance and contextual factors, such as emotions, social support and values, all of which should be taken into account when making recommendations and decisions in a court of law.

In addition to examining the ability of financial judgment in adults with ADHD, the current paper also determined the convergent and divergent validity of the financial judgment tests that were used. It is not surprising, considering the results described above, that the FCAI and FDMI do not lead to similar conclusions and have an insufficient convergent validity. This conclusion is corroborated by the small to medium correlations between the FCAI and FDMI as well as the no to slight agreement between the FCAI and FDMI and the fair agreement between the two vignettes of the FDMI. These findings related to convergent validity clearly illustrate one of the current points of discussion in the field of the assessment of financial capability, including financial judgment, which is that we deal with a multidimensional construct that can be operationalized and conceptualized in different ways (Engel et al. 2016; Ghesquiere et al. 2019). What is important to note in this context is, however, that the divergent validity of the financial judgment tests that were used in the present study was found to be sufficient. Also, a previous study conducted in a normal aging population demonstrated that correlations between performances on financial competence (including financial judgment), financial performance tests and standard neuropsychological tests were of a relatively small size (Bangma et al. 2017). Taken together, these findings indicate that (1) the FCAI and FDMI measure constructs that are not measured by other financial capability tests and that (2) standard neuropsychological tests cannot be used to evaluate financial judgment. This exemplifies the value and relevance of tests such as the FCAI and FDMI for both, clinical practice as well as research, and indicates that only using such specialized tests, are we able to assess the financial competence of adults with ADHD.

This study has some limitations that need to be taken into account when interpreting the results. First, a relatively large number of students was included in the group of adults without ADHD. Students are often in a special situation with regard to their finances since they usually live on loans or study grants. Even though it is not likely that this special financial situation causes students to perform differently on the tests that were used in the present study than non-students, it cannot be excluded. Second, adults with ADHD with comorbid disorders such as substance dependency and depressive disorder were not excluded from the current sample. Furthermore, adults with ADHD were assessed while being off stimulant medication for 48 h. It is unclear what the effects of comorbid disorders and the use of stimulant medication on performances on tests of financial judgment are. Third, the FDMI evaluates understanding, appreciation, reasoning and communication on a scale that ranges from 0 to 2. Scores of 1 or lower were, therefore, considered clinically relevant and similar to the clinical evaluation of the FCAI which classifies individuals as scoring ≦ 1 SD below the mean or as > 1 SD below the mean. The clinical relevance of scores 1 or lower on the FDMI has, however, not been determined and needs further exploration.

In conclusion, adults with ADHD have difficulties with financial judgment especially with the ability to understand information that is relevant for a financial situation or transaction. Furthermore, adults with ADHD were found to have problems with appreciating, reasoning and communicating about practical information that partly relates to their own financial situation. These difficulties with financial judgment of adults with ADHD are alarming as they can result in serious financial problems. A careful assessment of the financial capability of adults with ADHD, therefore, appears warranted in clinical practice. For this purpose, it is recommended to use tests such as the FCAI and FDMI that were particularly developed for the assessment of financial competence (including financial judgment) since these tests measure a construct that is not measured by standard (neuro)psychological tests. Furthermore, to get a complete overview of an individual's financial capability, it is advised to assess financial judgment, financial knowledge, financial performance as well as related contextual factors (Appelbaum et al. 2016; Engel et al. 2016). Finally, considering the results of this study, the development of teaching and coaching programs that support adults with ADHD in dealing with their financial matters are urgently needed, especially considering the importance of financial capability for individual autonomy.
